# Targeting the gut microbiota: the application and prospects of probiotics, fecal microbiota transplantation, and natural products in MASLD

**DOI:** 10.3389/fnut.2026.1735669

**Published:** 2026-02-24

**Authors:** Mengtian Li, Haoyu Zhai, Liping Qiao, Zhaobo Wang, Liyang Yang, Xinrui Zheng, Haoran Shi, Wenwen Geng, Jia Wang

**Affiliations:** 1 Department of General Internal Medicine, Guang’anmen Hospital, China Academy of Chinese Medical Sciences, Beijing, China; 2 Department of Oncology, Guang’anmen Hospital, China Academy of Chinese Medical Sciences, Beijing, China; 3 Jiangsu Provincial Hospital of Traditional Chinese Medicine Affiliated to Nanjing University of Chinese Medicine, Nanjing, China; 4 Department of Endocrinology, Guang’anmen Hospital, China Academy of Chinese Medical Sciences, Beijing, China; 5 Beijing University of Chinese Medicine, Beijing, China; 6 College of Traditional Chinese Medicine, Beijing University of Chinese Medicine, Beijing, China; 7 Traditional Chinese Medicine Department, The Fifth Affiliated Hospital of Zhengzhou University, Zhengzhou, China

**Keywords:** GM, gut-liver axis, MASLD, probiotics, FMT, natural products

## Abstract

Metabolic dysfunction-associated steatotic liver disease (MASLD) has emerged as the most prevalent chronic liver condition globally. Studies have revealed distinct differences in the gut microbiota (GM) composition between healthy individuals and MASLD patients, suggesting a crucial role of GM in disease initiation and progression. This review summarizes characteristic gut microbial alterations in MASLD, examines the relationship between GM and their metabolites in MASLD pathogenesis, and discusses potential mechanistic pathways. Furthermore, we summarize the possible therapeutic applications of probiotics, fecal microbiota transplantation (FMT), and natural products in managing MASLD through GM modulation. Although current evidence indicates these interventions may slow or prevent MASLD progression, most research remains limited to animal experiments and small-scale clinical studies. The scarcity of high-quality clinical evidence has created a significant gap between theoretical research and clinical application. Therefore, this article aims to summarize existing findings, explore the prospects of GM-targeted strategies for MASLD treatment, and propose future research directions in this field.

## 1 Introduction

Non-alcoholic fatty liver disease (NAFLD) is the most common cause of chronic liver disease globally, affecting up to 38% of the adult population worldwide as of 2023 (1). The global prevalence among adolescents also increased from 3.73% in 1990 to 4.71% in 2019, representing a relative increase of 26.27% (2). Its main feature is hepatic lipid accumulation resulting from insulin resistance and associated metabolic dysfunction (3). With growing understanding, NAFLD is now recognized as a multisystem disorder closely linked to metabolic syndrome, involving multiple extrahepatic organs and regulatory pathways (4). Consequently, in 2023, three major international liver associations recommended replacing the term “NAFLD” with “MASLD” (5). Metabolic dysfunction-associated steatotic liver disease (MASLD) can progress to an inflammatory stage known as metabolic dysfunction-associated steatohepatitis (MASH) and further advance to fibrosis, cirrhosis, and hepatocellular carcinoma (HCC). Although extensive studies have investigated the etiology of MASLD, its precise pathogenesis remains incompletely understood. In recent years, the gut microbiota (GM) has attracted increasing attention for its potential role in MASLD development.

The GM comprises trillions of bacteria, fungi, and archaea that play essential roles in maintaining host metabolic homeostasis. Evidence from both animal and human studies suggests that GM dysbiosis may be a key driver of MASLD pathogenesis (6, 7). Advances in high-throughput sequencing technologies, particularly shotgun metagenomic sequencing, have revealed strong associations between GM alterations and MASLD progression. For instance, increased levels of *Escherichia coli* and *Bacteroides vulgatus* are associated with advanced fibrosis in MASLD patients (8). A 2024 Mendelian randomization study further supported the potential causal relationship between specific gut bacterial taxa and MASLD development in humans (9).

The GM is highly plastic, and its compositional shifts can alter microbial metabolites, ultimately influencing host physiology through the gut–liver axis (10). Given its central role in MASLD, targeting the GM represents a promising therapeutic strategy. For instance, probiotic supplementation has been shown to improve liver enzymes, liver stiffness measurement (LSM), and hepatic steatosis in patients with MASLD (11). Fecal microbiota transplantation (FMT), an emerging therapeutic approach, has shown promising results in animal models and preliminary human trials (12). In high-fat diet (HFD)-induced MASLD mouse models, FMT reduced hepatic lipid accumulation and pro-inflammatory cytokine levels, and it also alleviated hepatic fibrosis and inflammatory infiltration in mice with established steatohepatitis (13). Numerous preclinical studies have shown that natural products can ameliorate MASLD by modulating the GM (14). However, outcomes across studies remain heterogeneous, with endpoints including liver enzyme levels and histological features such as steatosis, lobular inflammation, and fibrosis (15). Evidence supporting the therapeutic efficacy of FMT in MASLD remains limited (12). Moreover, although many natural products appear to alleviate MASLD, their mechanisms of action are complex, and the specific contribution of GM modulation to their beneficial effects remains unclear.

**Figure 1:**
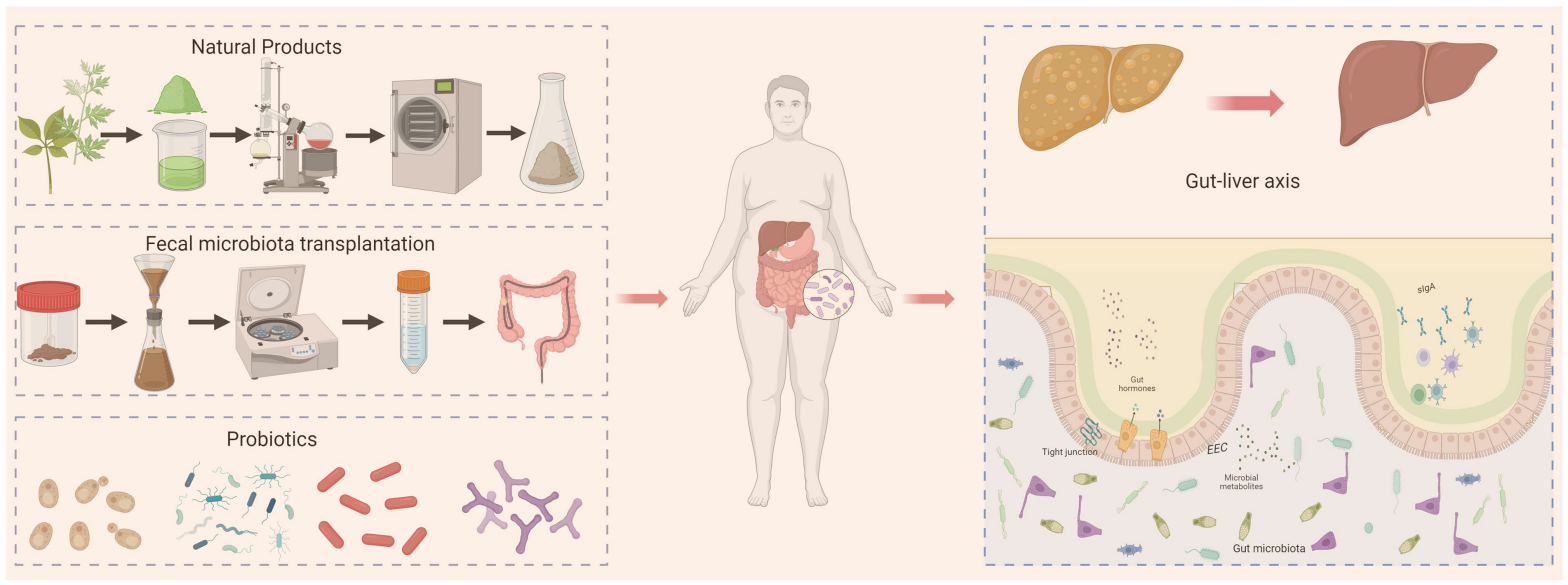
GM-targeted therapeutic strategies for MASLD, including probiotics, FMT, and natural products.

This review summarizes the current literature on MASLD, emphasizing the mechanisms by which the GM contributes to disease onset and progression. We discuss GM-targeted therapeutic strategies, including probiotics, FMT, and natural products. Furthermore, we highlight the existing gap between preclinical research and clinical application, and offer key perspectives on future directions. Ultimately, this review aims to deepen the understanding of the GM as a therapeutic target for MASLD and to provide a theoretical foundation and practical guidance for its clinical translation (Figure 1).

## 2 The role of GM in MASLD

### 2.1 GM signatures in MASLD

The GM plays a crucial role in fermenting dietary fibers, regulating immunity, maintaining intestinal barrier integrity, and synthesizing vitamins (16). Abnormal alterations in GM composition and function are implicated in the onset and progression of MASLD via the “gut–liver axis.” Since the GM is predominantly bacterial, most studies to date have focused on bacteria. The human GM is predominantly composed of the phyla Firmicutes, Bacteroidetes, Actinobacteria, Proteobacteria, Fusobacteria, and Verrucomicrobia, with Firmicutes and Bacteroidetes together accounting for over 90% of the total population (17). Within the Firmicutes phylum, dominant genera include *Ruminococcus*, *Blautia*, *Eubacterium*, and *Faecalibacterium*. The key genera within the Bacteroidetes phylum include *Bacteroides* and *Prevotella* (18), and their relative abundances are inversely correlated (19, 20). Actinobacteria, which constitute a relatively low proportion of the GM, are primarily represented by the genus *Bifidobacterium* (20).

The diversity and abundance of gut microorganisms are essential for maintaining intestinal homeostasis and function, thereby establishing a state of symbiotic equilibrium (21). However, numerous studies have shown that the GM composition in MASLD patients differs from that of healthy individuals. A recent meta-analysis of 54 studies found that MASLD patients exhibit significantly lower GM diversity than healthy controls (22). At the phylum level, the relative abundance of Bacteroidetes is generally lower in MASLD patients, although considerable heterogeneity across studies exists. As the disease progresses, the abundance of Proteobacteria gradually increases, while that of Firmicutes decreases (23–25). However, changes within the Firmicutes phylum are complex, with genera including *Eubacterium* and *Faecalibacterium* showing higher abundance in healthy controls, whereas *Enterococcus* and *Veillonella* were enriched in MASLD patients (24). At the family level, consistent reductions are observed in Ruminococcaceae (26, 27) and Prevotellaceae (28, 29). Similarly, at the genus level, studies consistently report a marked decrease in the anti-inflammatory genus *Faecalibacterium* (26, 30) alongside a consistently reported increase in the potentially pathogenic *Escherichia–Shigella* group (24, 28). However, reported abundances of key genera such as *Prevotella* and *Bacteroides* vary across studies (23, 26, 31). These inconsistencies may be related to the stage of disease progression and the geographic distribution of the population (32). For instance, in a study of pediatric MASLD, a high abundance of *Prevotella copri* was associated with more severe liver fibrosis and was correlated with lower α-diversity and Hispanic ethnicity (33). Enrichment of the *Escherichia–Shigella* group within the Proteobacteria phylum is observed in both pediatric and adult MASH patients (28, 34), accompanied by the depletion of beneficial genera such as *Ruminococcus obeum* and *Eubacterium rectale* (8). Given the close association between MASLD and obesity, researchers have examined potential GM differences between obese and non-obese patients. Despite controlling for obesity-related confounders, the reduction of *Faecalibacterium* and *Ruminococcus* persists, suggesting that these changes may be independent of obesity (35, 36).

Collectively, alterations in the GM are closely associated with the pathogenesis of MASLD, although considerable heterogeneity exists across studies. MASLD is characterized by reduced GM diversity, diminished microbial abundance, depletion of anti-inflammatory taxa, and enrichment of pro-inflammatory species, although specific microbial changes are inconsistent across studies. Apart from the factors already mentioned, dietary composition and habits also shape the GM and may contribute to MASLD progression (37). Moreover, the predominant reliance on fecal samples, which represent the luminal content of the distal colon, does not fully capture the microbial composition at other intestinal sites, such as the small intestine or mucosal surfaces. Future studies employing segmental sampling along the gastrointestinal tract are warranted to provide a spatially resolved and more comprehensive understanding of GM alterations in MASLD.

### 2.2 Pathogen-associated molecular patterns (PAMPs) and MASLD

The gut and liver communicate bidirectionally via the gut–liver axis. Within this axis, the synthesis and secretion of bile by the liver facilitate direct communication with the intestine via the biliary system, thereby contributing to the maintenance of GM homeostasis (38). Microorganisms in the gut metabolize endogenous substrates (e.g., bile acids and amino acids derived from protein breakdown) and exogenous substrates (derived from the diet and environmental exposures), generating metabolites that enter the liver via the portal vein. The liver further processes these compounds and releases inflammatory mediators or metabolites into the systemic circulation, which may, in turn, influence intestinal function (39). The integrity of the intestinal barrier is essential for the proper function of this axis. It operates through a three-tiered defense system that prevents the translocation of pathogens and harmful substances into the systemic circulation (40).

The immune barrier relies on intestinal epithelial cells, gut-associated lymphoid tissue (GALT), and immune cells to achieve dynamic immunoregulation. Intestinal epithelial cells not only express anti-inflammatory cytokines such as interleukin 10 (IL-10) (41), but also express various pattern recognition receptors (PRRs), including Toll-like receptors (TLRs) and nucleotide-binding oligomerization domain (NOD) proteins. Upon recognizing microbial signals, these receptors can induce the production of chemokines (42). GALT comprises Peyer’s patches (PPs) and isolated lymphoid follicles (ILFs), which are aggregates of lymphoid cells including dendritic cells, T cells, regulatory T cells (Tregs), and B cells (43). In the PPs-associated epithelium, specialized microfold cells (M cells) efficiently take up and transport luminal antigens, a process that may involve receptors such as TLRs. Subsequently, M cells facilitate the secretion of cytokines and chemokines, thereby activating underlying immune cells and inducing the generation of secretory immunoglobulin A (SIgA)-producing plasma cells. These sites are key locations for triggering adaptive immune responses, and the produced SIgA serves as a major effector molecule in intestinal tissues (44, 45). The mechanical barrier consists of epithelial cells—including absorptive enterocytes, goblet cells, enteroendocrine cells, Paneth cells, and M cells—together with intercellular junctions and the mucus layer (46). Epithelial cells form a selectively permeable interface through tight junction proteins such as occludin and zonula occludens-1 (ZO-1), as well as adherens junctions, desmosomes, and gap junctions (47). Goblet cell-secreted mucins, such as MUC2 and MUC5AC, form the mucus layer, which protects intestinal cells from external factors (48). The mucus layer also plays an important role in the interaction with the GM, providing nutrients and attachment sites (49). The biological barrier comprises the GM, which constitutes a dynamic and symbiotic ecosystem. Under physiological conditions, the GM and its metabolites exert beneficial effects by preventing pathogen invasion and maintaining intestinal microenvironmental homeostasis (50). However, in MASLD, gut dysbiosis disrupts intestinal barrier integrity, thereby exacerbating disease progression.

Studies have shown that increased intestinal permeability is highly prevalent among patients with MASLD. A meta-analysis including 128 MASLD patients revealed that approximately 39.1% exhibited elevated intestinal permeability, as assessed by the urinary excretion of permeability markers, compared to only 6.8% in healthy controls. Notably, nearly 49.2% of patients with MASH were found to have increased intestinal permeability (51). GM dysbiosis is a key contributor to this impairment of the intestinal barrier. Evidence from an FMT study demonstrated that germ-free mice receiving feces from HFD—fed mice developed increased intestinal permeability, whereas those receiving feces from standard diet-fed mice did not. This finding indicates that HFD-induced dysbiosis acts as a causal factor in intestinal barrier impairment (52). However, the mechanisms underlying GM alterations that lead to increased intestinal permeability and impaired barrier integrity remain largely unclear. One plausible explanation is that GM dysbiosis in MASLD directly downregulates the expression of tight junction proteins, such as ZO-1 and occludin (53, 54), thereby disrupting the tight junction structure (55). Moreover, ethanolamine serves as both an essential phospholipid component of mammalian cell membranes and an energy source for several bacterial species (56, 57). A recent study further demonstrated that in obese mouse models, the levels of ethanolamine-metabolizing GM are significantly reduced, leading to the accumulation of ethanolamine in the gut (58). This elevated ethanolamine was shown to upregulate microRNA-101a-3p expression, which decreases the mRNA stability of tight junction proteins, suppresses their translation, and ultimately compromises intestinal barrier integrity.

The disruption of the intestinal barrier, driven by GM dysbiosis, facilitates the translocation of PAMPs. Among these, the translocation of lipopolysaccharide (LPS), a component of the outer membrane of Gram-negative bacteria, is particularly notable (59). Patients with MASLD exhibit significantly higher serum LPS levels than healthy individuals, and these levels correlate positively with MASLD disease stages, indicating that LPS serves as a key promoter of disease progression (60). Mechanistically, LPS contributes to MASLD pathogenesis through multiple pathways. First, LPS has been demonstrated to directly increase intestinal epithelial permeability by disrupting tight junctions under both experimental and physiological conditions (61). Second, LPS, which is commonly referred to as endotoxin, translocates to the liver via the portal vein and promotes hepatic steatosis progression (62).

**Figure 2:**
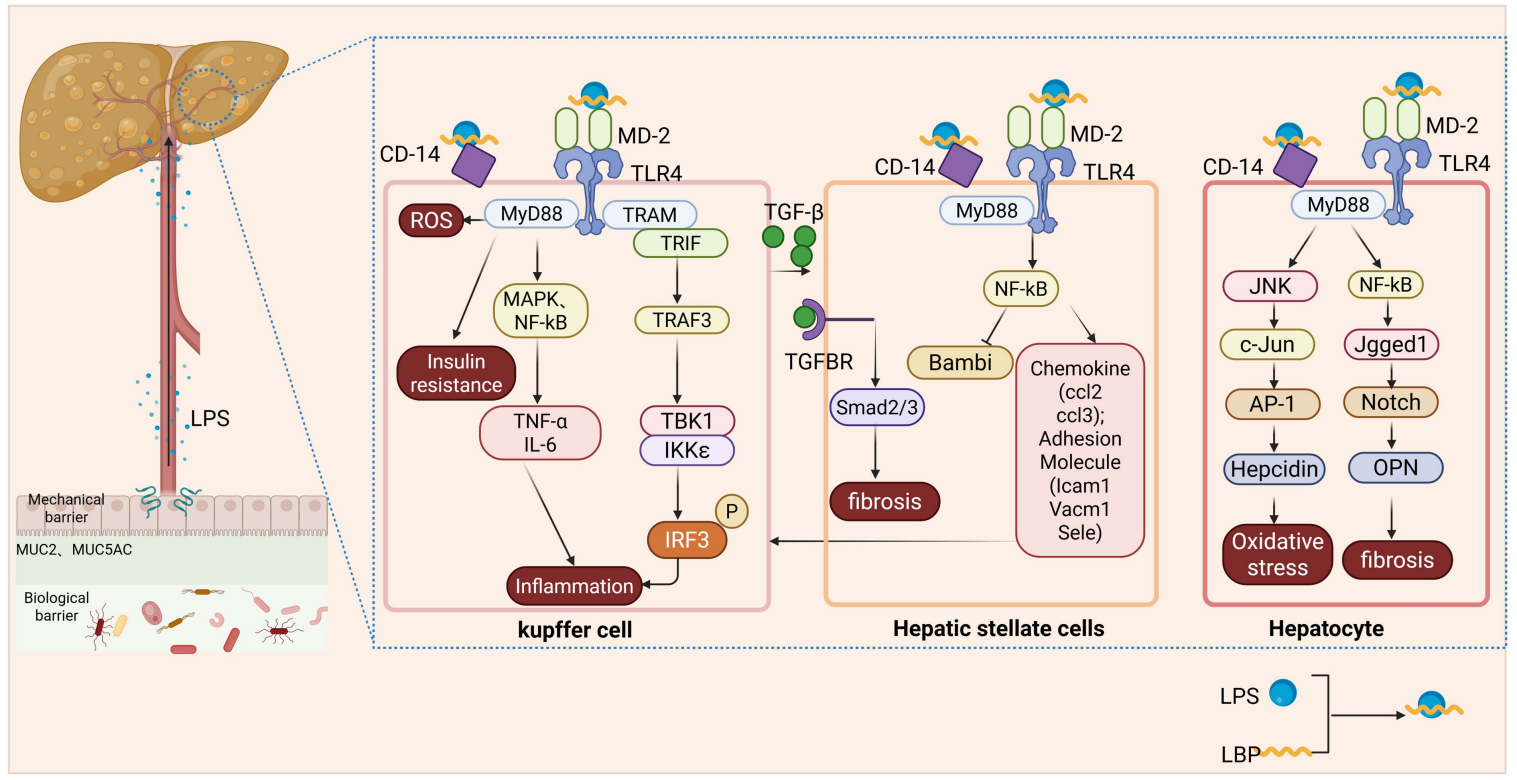
Pathological cascade triggered by intestinal barrier disruption in MASLD. Gut dysbiosis and increased intestinal permeability facilitate the translocation of PAMPs, particularly LPS, from the gut lumen into the portal circulation. Upon reaching the liver, LPS activates various cell types via the TLR4 signaling pathway. In Kupffer cells, the LPS-LBP complex binds to the CD14/TLR4/MD-2 receptor, initiating either the MyD88-dependent or TRIF-dependent signaling pathways, which drive hepatic inflammation and insulin resistance. In HSCs, LPS binding enhances TGF-β signaling by downregulating the pseudoreceptor BAMBI, thereby promoting HSC activation and collagen production and driving liver fibrosis. Activated HSCs also secrete chemokines to recruit Kupffer cells. In hepatocytes, TLR4 activation induces hepcidin expression via the JNK/c-Jun/AP-1 pathway, exacerbating oxidative stress, and upregulates OPN via the Jagged1-Notch pathway, further promoting liver fibrosis.

At the molecular level, LPS binds to lipopolysaccharide-binding protein (LBP) in the liver. This complex then facilitates the activation of the Toll-like receptor 4 (TLR4)/myeloid differentiation factor-2 (MD-2) complex on Kupffer cells, with the assistance of cluster of differentiation 14 (CD14), thereby initiating downstream signaling cascades. TLR4 activation influences MASLD pathogenesis primarily through two distinct pathways: the myeloid differentiation primary response 88 (MyD88)-dependent pathway and the TIR-domain-containing adapter-inducing interferon-β (TRIF)-dependent pathway. In the MyD88-dependent pathway, TLR4 recruits the adaptor protein MyD88, which activates mitogen-activated protein kinase (MAPK) and nuclear factor-kappa B (NF-κB) signaling cascades. This activation promotes the expression of pro-inflammatory cytokines, such as tumor necrosis factor-alpha (TNF-α), IL-6, IL-8, and IL-12, as well as chemokines including interferon-gamma (IFN-*γ*) and monocyte chemoattractant protein-1 (MCP-1), thereby driving inflammation (63). This pathway in Kupffer cells also contributes to hepatic reactive oxygen species (ROS) formation and insulin resistance (64). In the MyD88-independent/TRIF-dependent pathway, the adaptor proteins TRIF and the TRIF-related adaptor molecule (TRAM) recruit TNF receptor-associated factor 3 (TRAF3). TRAF3 subsequently activates IKK-related kinases, including TANK-binding kinase 1 (TBK1) and Inhibitor of kappa B (IκB) kinase epsilon (IKKε), resulting in the phosphorylation of interferon regulatory factor 3 (IRF3). The phosphorylated IRF3 then dimerizes and translocates into the nucleus, where it induces the expression of interferon-stimulated genes and inflammatory mediators (65). In hepatocytes, LPS binding to TLR4 activates the MyD88-dependent signaling pathway. This activation triggers a downstream cascade involving Interleukin-1 Receptor-Associated Kinase 1 (IRAK1) and TNF Receptor-Associated Factor 6 (TRAF6), leading to the specific activation of c-Jun N-terminal kinase (JNK). Activated JNK subsequently phosphorylates the transcription factor c-Jun, which forms the activator protein-1 (AP-1) complex and drives the transcription of hepcidin. The resulting disruption of iron metabolism exacerbates oxidative stress and promotes lipid peroxidation in hepatocytes, thereby contributing to MASLD progression (66). Activated Notch signaling upregulates osteopontin (OPN), which acts on hepatic stellate cells (HSCs) to promote their activation and the subsequent development of hepatic fibrosis (67). LPS also interacts directly with quiescent HSCs, leading to marked downregulation of bone morphogenetic protein (BMP) and the activin membrane-bound inhibitor (BAMBI), a pseudoreceptor for TGF-β. This downregulation relieves the intrinsic inhibition of TGF-β signaling, thereby sensitizing HSCs to TGF-β stimulation. Sensitized HSCs secrete abundant chemokines (e.g., CCL2 and CCL3) and express adhesion molecules such as intercellular adhesion molecule-1 (ICAM-1), vascular cell adhesion molecule-1 (VCAM-1), and E-selectin, actively recruiting Kupffer cells to their vicinity. These recruited Kupffer cells, in turn, release active TGF-β, amplifying TGF-β signaling and promoting the transdifferentiation of HSCs into myofibroblasts, thereby driving hepatic fibrosis (68) (Figure 2).

### 2.3 GM-derived metabolites and MASLD

**Figure 3:**
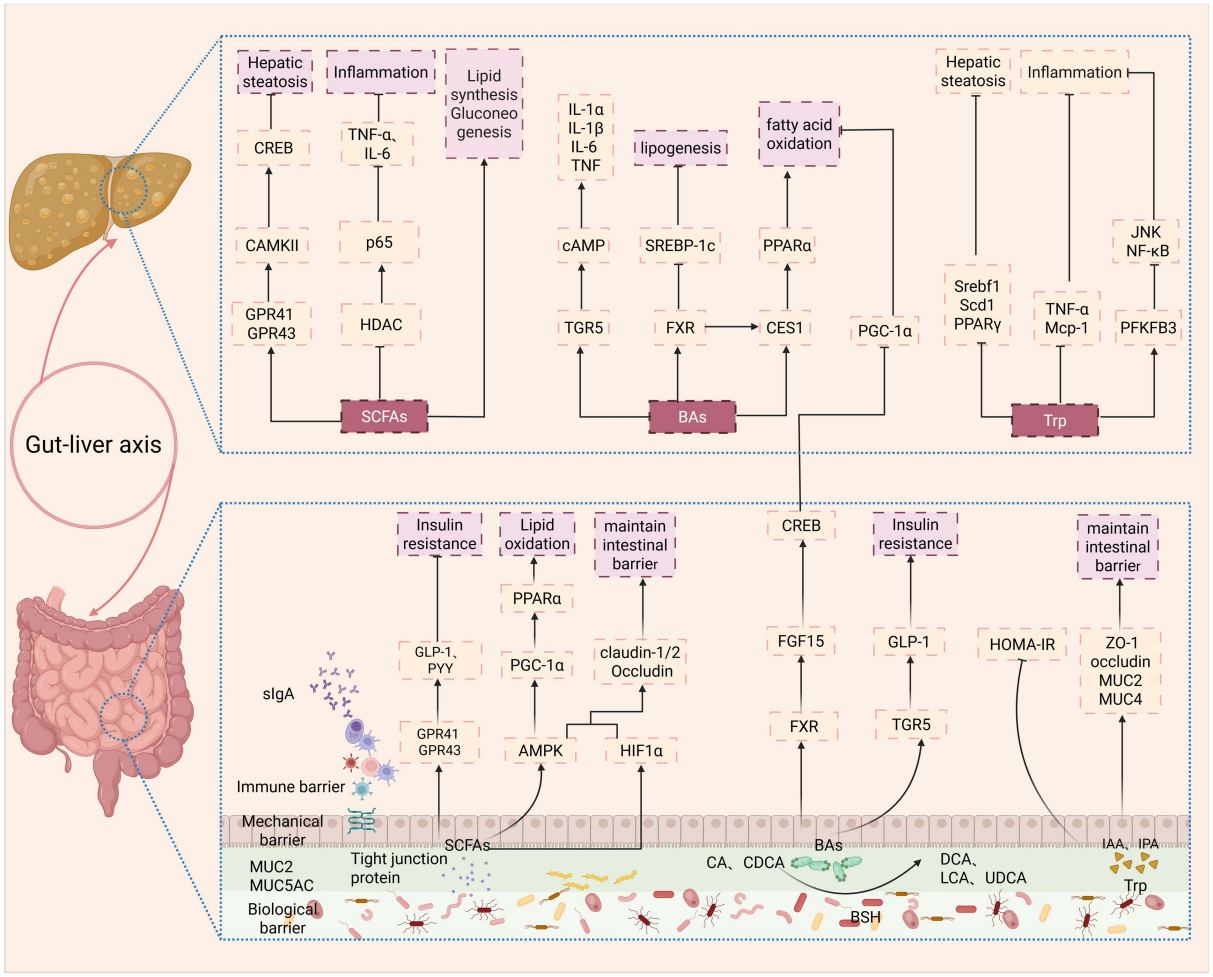
This figure illustrates the dual roles of key GM-derived metabolites, SCFAs, BAs, and tryptophan derivatives in MASLD. SCFAs: Their effects are context-dependent. They primarily exert protective effects by activating AMPK and G protein-coupled receptors (e.g., GPR41/43), leading to enhanced lipid oxidation, improved insulin sensitivity, strengthened intestinal barrier, and attenuated inflammation. Conversely, they can also serve as substrates for *de novo* lipogenesis in the liver, potentially promoting hepatic steatosis. BAs: The GM converts primary BAs to secondary BAs, thereby altering signaling through receptors such as the FXR and TGR5. This process regulates BA homeostasis, lipid metabolism, and inflammation. MASLD is associated with an altered BA pool, which disrupts these pathways and promotes steatosis and inflammation. Tryptophan derivatives (e.g., IAA and IPA): These metabolites exhibit protective effects by improving insulin sensitivity, reducing lipogenesis, strengthening the intestinal barrier, and inhibiting inflammatory pathways such as NF-κB.

In addition to the translocation of PAMPs such as LPS, metabolites produced by the GM also play a crucial role in the pathogenesis and progression of MASLD. These metabolites act as key mediators of host–microbiota crosstalk and play a major role in hepatic metabolic and immune regulation via the gut–liver axis. Among the wide array of microbial metabolites, the most extensively studied classes in the context of MASLD include short-chain fatty acids (SCFAs), bile acids (BAs), and tryptophan (Trp) derivatives (Figure 3).

#### 2.3.1 SCFAs

SCFAs, primarily acetate, propionate, and butyrate, are bioactive metabolites generated through microbial fermentation of dietary fiber and resistant starches in the colon (69). The concentration of SCFAs in MASLD patients varies across different studies (70–72). SCFAs play a dual role in MASLD pathophysiology, as they can either promote or inhibit disease progression.

Typically, SCFAs exert protective effects against MASLD by promoting energy expenditure and lipid oxidation (73). A 24-week intervention study in overweight individuals showed that the targeted colonic delivery of propionate significantly reduced hepatic lipid content (74). Multiple animal studies have indicated that SCFA supplementation ameliorates hepatic steatosis and inflammation in MASLD mice by modulating hepatic lipid metabolism in an adenosine monophosphate (AMP)-activated protein kinase (AMPK)-dependent manner (75). Specifically, GM-derived acetate activates the AMPK–peroxisome proliferator-activated receptor gamma (PPARγ) coactivator 1-alpha (PGC-1α)–peroxisome proliferator-activated receptor alpha (PPARα) pathway, which suppresses chylomicron secretion in enterocytes and enhances lipid oxidation (76). SCFAs also act through the activation of G protein-coupled receptors, such as G-protein-coupled receptor 41 (GPR41) and GPR43. In the liver, butyrate supplementation activates the GPR41/43-mediated calcium/calmodulin-dependent protein kinase II–(CaMKII)–cyclic adenosine monophosphate (cAMP) response element-binding protein (CREB) pathway in hepatocytes, thereby inhibiting hepatic steatosis and promoting fatty acid oxidation in mice (77).

In the gut, GPR41 and GPR43 stimulate L cells to secrete glucagon-like peptide-1 (GLP-1) and peptide YY (PYY), thereby improving insulin resistance (78). Furthermore, SCFAs, especially butyrate, significantly enhance intestinal barrier integrity (79). This effect involves AMPK-mediated upregulation of claudin-2 (80), and HIF1α-driven expression of claudin-1 and occludin (81). Interestingly, SCFAs also appear to epigenetically regulate MASLD progression. Butyrate has been shown to inhibit histone deacetylase (HDAC) activity in mice, thereby increasing acetylation of the NF-κB subunit p65. This modification reduces the expression of pro-inflammatory genes and promotes apoptosis of pro-inflammatory macrophages (82). Additionally, studies in cultured mouse macrophage cell lines have demonstrated that SCFAs attenuate LPS-induced production of TNF-α, IL-1β, and IL-6 while enhancing LPS-stimulated IL-10 production (83). However, SCFAs may also contribute to the development of MASLD. Absorbed SCFAs delivered via the portal vein can serve as direct substrates for hepatic lipogenesis and gluconeogenesis, thereby promoting intrahepatic lipid accumulation (84). Studies have shown that GPR43-deficient mice exhibit lower hepatic triglyceride levels (85). Similarly, under HFD conditions, intestine-specific GPR41 knockout mice display decreased body weight and fat mass (86). These findings indicate that activation of these GPRs may promote obesity and metabolic dysfunction through multiple mechanisms, thereby contributing to the pathogenesis of fatty liver disease.

In conclusion, future research should focus on elucidating the distinct roles of individual SCFAs in MASLD and on exploring novel therapeutic strategies that precisely regulate specific SCFA levels.

#### 2.3.2 BAs

Another important function of the GM is converting primary BAs into secondary BAs. BAs are derived from cholesterol. Free BAs are conjugated with taurine or glycine in the liver (87), stored in the gallbladder, and released into the intestine upon food intake (88). Approximately 75% of primary BAs are synthesized through the classical pathway in hepatocytes, a process catalyzed by cholesterol 7α-hydroxylase (CYP7A1) (89, 90). Approximately 5% of primary BAs (e.g., cholic acid [CA] and chenodeoxycholic acid [CDCA]) are hydrolyzed by bacterial bile salt hydrolase (BSH) and subsequently converted by gut anaerobes via 7α-dehydroxylase into secondary bile acids, such as deoxycholic acid (DCA) and lithocholic acid (LCA). In contrast, about 95% of BAs are reabsorbed in the terminal ileum via the apical sodium-dependent bile acid transporter (ASBT) and returned to the liver (91). The remaining BAs excreted in feces represent the primary route of cholesterol elimination and are essential for maintaining systemic steroid homeostasis. BAs act as potent signaling molecules that regulate their own synthesis, glucose and lipid metabolism, and inflammatory responses by modulating nuclear receptors such as the farnesoid X receptor (FXR) and the Takeda G protein-coupled receptor 5 (TGR5) (92).

FXR serves as a master nuclear receptor that regulates BA metabolism and lipid homeostasis (93, 94). It modulates BA synthesis through two distinct pathways. In the liver, BA-activated FXR induces small heterodimer partner (SHP), which inhibits the transcriptional activity of hepatocyte nuclear factor 4α (HNF4α) and liver receptor homolog-1 (LRH-1), thereby directly suppressing the expression of the rate-limiting BA synthetic enzymes CYP7A1 and sterol 12α-hydroxylase B1 (CYP8B1) (92). In the ileum, BA-activated FXR induces fibroblast growth factor 15 (FGF15; its human ortholog is FGF19) (95). FGF15/19 is secreted into the portal circulation and activates the hepatic FGFR4/β-Klotho complex, triggering downstream signaling, including the extracellular signal-regulated kinase (ERK) pathway, thereby indirectly suppressing CYP7A1 (96). Notably, selective activation of intestinal FXR through the aforementioned enteric pathway elicits a spectrum of metabolic improvements that extend beyond mere inhibition of BA synthesis, offering a unique advantage in alleviating hepatic steatosis. The core mechanism involves the intestinal FXR-FGF15/19 axis-mediated inhibition of CYP7A1, altering the composition of the BA pool and leading to a relative increase in the proportion of secondary BAs such as LCA. By activating the widely expressed TGR5 in adipose tissue, LCA stimulates thermogenesis in brown adipose tissue (BAT) and induces the “browning” of white adipose tissue (WAT), thereby significantly enhancing systemic energy expenditure and improving insulin sensitivity, which collectively contribute to the amelioration of hepatic steatosis (97). Concurrently, hepatic FXR activation directly mitigates steatosis through dual mechanisms: suppression of sterol regulatory element-binding protein 1c (SREBP-1c) reduces *de novo* lipogenesis (98), while upregulation of carboxylesterase 1 (CES1) promotes fatty acid release and subsequent PPARα-driven fatty acid oxidation, thereby decreasing hepatic lipid accumulation (99, 100). However, the FXR signaling network exhibits context-dependent effects. For instance, studies have found that under specific conditions, the intestinal FXR-FGF15 axis may suppress hepatic cAMP response element-binding protein (CREB), leading to downregulation of hepatic PGC-1α expression and subsequently inhibiting fatty acid oxidation (101, 102). This further underscores the importance of precisely regulating tissue-specific FXR activity. Collectively, FXR orchestrates systemic energy metabolism and hepatic lipid homeostasis through the aforementioned multi-tissue, multi-layered, and coordinated mechanisms.

Indeed, BAs also exert multifaceted beneficial effects in ameliorating MASLD by activating TGR5. Specifically, BAs promote GLP-1 secretion in obese mice, thereby enhancing insulin sensitivity (103), and concurrently suppress the activation of the inflammatory transcription factor NF-κB (104). Furthermore, BAs can inhibit LPS-induced cytokine production—including interleukin-1α (IL-1α), IL-1β, IL-6, and tumor necrosis factor (TNF)—via a TGR5–cAMP-dependent pathway in Kupffer cells, directly mitigating hepatic inflammation (105). At the intestinal level, activation of FXR and TGR5 by BAs in intestinal epithelial cells enhances tight junction protein expression, improves barrier function, and ultimately reduces the portal influx of pro-inflammatory stimuli by restricting bacterial translocation (106, 107).

In MASLD, GM-driven alterations in the BA pool modulate the activity of key receptors such as FXR and TGR5, thereby driving critical pathological processes, including hepatic steatosis, inflammation, insulin resistance, and fibrosis. Collectively, these findings suggest that targeting bile acid metabolism and receptor signaling represents a promising therapeutic strategy for MASLD.

#### 2.3.3 Tryptophan derivatives

Tryptophan (Trp) plays a pivotal role in metabolic regulation and serves as a precursor for multiple bioactive compounds. Under physiological conditions, approximately 4%–6% of dietary tryptophan is metabolized by the GM into various indole derivatives, including indole-3-acetic acid (IAA), indole-3-propionic acid (IPA), indole-3-lactic acid, indole-3-carboxylic acid, and tryptamine (108). Accumulating evidence indicates that indole derivatives play protective roles in MASLD progression. GM dysbiosis associated with MASLD significantly reduces the production of indoles and their derivatives in both animal models and humans (109, 110).

In HFD-induced MASLD mouse models, IAA significantly improves insulin resistance, as assessed by the homeostasis model assessment of insulin resistance (HOMA-IR). Moreover, IAA downregulates the expression of key lipogenic genes, such as sterol regulatory element-binding protein 1 (*Srebf1*), stearoyl-coenzyme A (CoA) desaturase 1 (*Scd1*), and peroxisome proliferator-activated receptor gamma (PPARγ), and reduces the expression of inflammatory mediators such as MCP-1 and TNF-α, thereby alleviating hepatic steatosis (111). IPA restores HFD-fed gut dysbiosis and enhances intestinal barrier integrity by upregulating tight junction proteins (ZO-1 and occludin) and mucins (MUC2 and MUC4). These effects collectively reduce LPS translocation and subsequent TLR4 signaling activation, ultimately mitigating MASH pathology (112, 113). Furthermore, IPA inhibits NF-κB signaling and decreases pro-inflammatory cytokines such as IL-1β and IL-6, thereby mitigating liver inflammation (112). *In vitro* studies using hepatocytes have shown that indole suppresses LPS-induced pro-inflammatory responses in macrophages by inhibiting JNK and NF-κB phosphorylation and reducing the release of inflammatory mediators. This effect may be mediated through the upregulation of 6-phosphofructo-2-kinase/fructose-2,6-bisphosphatase 3 (PFKFB3) in macrophages (109). In mice, oral indole administration following endotoxin challenge reduces prostaglandin-associated inflammatory cytokines (IL-1β, IL-6, and IL-15) and NF-κB expression, thereby ameliorating liver inflammation (114).

In summary, indole derivatives generated through gut microbial metabolism of tryptophan play a crucial role in maintaining metabolic homeostasis and attenuating MASLD progression. Therefore, targeting indole metabolism may represent a promising novel therapeutic strategy for MASLD.

In conclusion, the link between the GM and MASLD has become increasingly well established. Dysbiosis of the GM, disruption of the intestinal barrier, translocation of PAMPs, and dysregulation of microbial metabolites collectively drive the progression of MASLD. Although the precise mechanisms remain to be fully elucidated, current evidence provides a solid theoretical foundation for developing interventions targeting the GM.

## 3 Targeting GM for MASLD treatment: probiotics, FMT, and natural products

Building upon this evidence, GM dysbiosis is now recognized as a central driver of MASLD pathogenesis, making it a promising therapeutic target. Consequently, interventions aimed at restoring microbial homeostasis, reinforcing intestinal barrier function, and enhancing the production of beneficial microbial metabolites have attracted substantial attention. Among these approaches, probiotics, FMT, and natural products have been extensively investigated as therapeutic strategies for MASLD. Their therapeutic potential lies in their capacity to remodel the gut ecosystem, preserve intestinal barrier integrity, and modulate host metabolic and immune pathways. This section summarizes these three strategies, emphasizing their mechanisms of action, preclinical and clinical evidence, and translational challenges and future prospects.

### 3.1 Probiotics

Animal models of MASLD have demonstrated that probiotics can mitigate liver injury, improve metabolic parameters, and modulate the GM through multiple pathways (Table 1). *Lactobacillus* and *Bifidobacterium* are the most studied probiotic genera for MASLD treatment (39). Among them, *Lactobacillus kefiranofaciens* ZW3 was found to restore intestinal mucosal structure, reduce LPS translocation, and inhibit the pro-inflammatory TLR4/MyD88 signaling pathway (115). This probiotic activity may be linked to its ability to secrete exopolysaccharides, which play an important role in protecting the physical intestinal barrier and regulating immune responses (116). Disordered BA metabolism interacts with the GM, leading to intestinal barrier dysfunction. This elevates the risk of endotoxemia, which, in turn, activates the immune system and exacerbates inflammation, thereby driving MASLD progression (117). Targeting the FXR signaling pathway is a key therapeutic strategy for MASLD. In an animal study, *Lactobacillus* significantly activated hepatic FXR and upregulated FGF15 expression at both mRNA and protein levels. Concurrently, it reduced serum total cholesterol (TC), triglyceride (TG), and total BA levels. These findings suggest that this strain inhibits excessive BA synthesis and lipid deposition via the FXR–FGF15 axis (118). Another study demonstrated that *Bifidobacterium bifidum* from the BeNa Culture Collection of China alleviates MASLD by activating hepatic FXR while suppressing intestinal FXR. It also modulates GM composition and enhances intestinal barrier function (119). As key microbial metabolites, SCFAs influence energy metabolism and inflammation through diverse pathways. *Lactobacillus reuteri* DSM 17938 restores the physiological ratio of acetate, propionate, and butyrate, thereby suppressing inflammation (120). *Bifidobacterium longum* BL-19 specifically promotes butyrate production and modulates hepatic CYP7A1 activity, improving BA metabolism and reducing hepatic lipid deposition (121). Both *Bifidobacterium breve* CKDB002 and *Bifidobacterium longum* CKDB004 modulate GM and metabolites, improve intestinal barrier function, and attenuate MASLD. However, *B. longum* CKDB004 appears more effective in suppressing the proliferation of the harmful genus *Helicobacter* (122). This suggests functional differentiation among probiotic strains in regulating microbial communities.

**Table 1:** Preclinical studies of probiotics in MASLD/MASH models.

No.	Probiotics	Modeling	Intervention	Mechanism	Outcomes	GM changes	References
01	*Lactobacillus kefiranofaciens* ZW3	MCDdiet-induced rats	2.15 × 10^11^ CFU/day, 8 weeks	Modulation of GM, Repair of the intestinal barrier, and inhibition of inflammatory pathways (TLR4-MyD88 and JNK)	TC, TG ↓; ALT, AST ↓; NAS ↓; Claudin-1 ↑; LPS ↓; NF-κB, TNF-α, IL-1β, IL-6 ↓; IL-4, IL-10 ↑	F/B ↑; *Lactobacillus*, Lachnospiraceae_NK4A136_group, Ruminococcaceae_NK4A214_group ↑; *Escherichia–Shigella*, *Bacteroides*, *Parabacteroides* genus ↓	(115)
02	Compound Eosinophil-Lactobacillus Tablets	HFD-induced mice	312 mg/kg/day, 8 weeks	Modulation of GM, Activation of the hepatic FXR/FGF15 pathway, Improving BA and lipid metabolism	TC, TG ↓; ALT, AST, TBA ↓; NAS ↓; FXR mRNA, FGF15 ↑	Bacteroidia class ↓; Porphyromonadaceae family ↓; Desulfovibrionaceae family ↑	(118)
03	*Bifidobacterium bifidum* from the BeNa Culture Collection of China	HFD-induced mice	2 × 10^8^ CFU, three times per week, 8 weeks	Modulation of GM, Repair of the intestinal barrier, Regulation of FXR expression, and modulation of immune status	Body weight, liver weight, and liver index ↓; FBG, HOMA-IR ↓; serum ALT ↓; serum TG, TC ↓; TNF-α, IL-6, IL-17A ↓; Th17 cell proportion ↓; Treg cell proportion ↑; ZO-1, Occludin ↑; hepatic lipid deposition ↓	*Lactobacillus*, *Parabacteroides*, *Romboutsia*, *Clostridia* UCG-014, *Mycoplasma* genus ↑; *Adlercreutzia*, *Rikenellaceae*, *Sphingobacterium*, *Sulfitobacter*, *Alobaculum* genus ↓	(119)
04	*Lactobacillus reuteri* DSM 17938	HFD-induced mice	2 × 10^9^ CFU/day, 12 weeks	Modulation of GM, Inhibition of inflammation, Alleviation of oxidative stress, Activation of autophagy pathways, and Improvement of insulin resistance and metabolic disorders	NAS ↓; TC, TG, LDL ↓; ALT, AST ↓; HOMA-IR ↓; MDA ↓; LPS-TLR4, NF-κB, TNF-α ↓; p-AKT, mTOR ↓; SCFAs ↑	Firmicutes phylum ↑, Bacteroidetes phylum ↓	(120)
05	*Bifidobacterium longum* BL-19	HFD-induced mice	1 × 10^9^ CFU/day, 12 weeks	Modulation of GM, Promotion of intestinal butyrate production, Modulation of CYP7A1 expression and hepatic BA metabolism	TNF-α, NLRP3 mRNA ↓; IL-10 mRNA ↓; CYP7A1 mRNA ↑; serum ALT ↓; SOD ↑; MDA ↓; Fecal butyrate ↑	TM7, Verrucomicrobia phylum ↓; Acidobacteria, Firmicutes phylum ↑; *Bacteroides*, *Parabacteroides*, *Ruminococcus* genus ↓; *Anaerostipes*, *Fusobacterium*, *Clostridium* genus ↑	(121)
06	*Lactobacillus sakei* MJM60958	HFD-induced mice	1 × 10^9^ CFU/day, 12 weeks	Modulation of GM, Alleviation of inflammation and metabolic disorders, and Regulation of lipid metabolism genes	Body weight ↓, TC, TG, ALT, AST ↓; TNF-α, IL-1β ↓; hepatic lipid deposition ↓; Leptin, Adiponectin ↓; FAS, SREBP-1 ↓; PPARα ↑; Acetate ↑	Firmicutes, Actinobacteria phylum ↓, Verrucomicrobia, Tenericutes phylum ↑; Akkermansiaceae, Ruminococcaceae family ↑	(162)
07	Lactiplantibacillus plantarum DSM20174	HFFD-induced mice	1 × 10^9^ CFU/day, 10 weeks	Modulation of GM, Improvement of metabolic risk factors, and alleviation of white adipose tissue inflammation	Body weight ↓; TG, TC, NEFA ↓; FBG, HOMA-IR ↓; IL-1β, IL-6 ↓; IL-4, IL-5 ↑; PPAR*γ*, CD36, *FASN*, CPT1, TLR4, MCP-1 ↓; M1 macrophages ↓; M2 macrophages ↑	Christensenellaceae family ↓; *Christensenella*, *Phocaeicola* ↓; A*cetatifactor*, *Duncaniella*, *Monoglobus*, *Lawsonibacter* ↑; *Ruminococcus torques* species ↓	(163)

MCD, methionine-choline-deficient; ALT, alanine aminotransferase; AST, aspartate aminotransferase; NAS, non-alcoholic fatty liver disease (NAFLD) activity score; F/B, Firmicutes/Bacteroidetes ratio; FBG, fasting blood glucose; p-AKT, phosphorylated Ak strain transforming; MDA, malondialdehyde; mTOR, mechanistic target of rapamycin; NLRP3, activation of the NACHT, LRR, and PYD domains-containing protein 3; SOD, superoxide dismutase; FAS, fatty acid synthase; SREBP-1, sterol regulatory element-binding protein 1; HFFD, high-fat high-fructose diet; NEFA, non-esterified fatty acids; CD36, cluster of differentiation 36; FASN, fatty acid synthase; CPT1, carnitine palmitoyl transferase 1; CFU, colony-forming unit.

Despite strong preclinical evidence, the efficacy of probiotics in MASLD patients shows heterogeneity (Table 2). A randomized double-blind placebo-controlled trial reported that a probiotic mixture containing six strains from *Bifidobacterium* and *Lactobacillus* (*Lactobacillus acidophilus* BCMC^®^ 12130, *Lactobacillus casei* BCMC^®^ 12313, *Lactobacillus lactis* BCMC^®^ 12451, *B. bifidum* BCMC^®^ 02290, *Bifidobacterium infantis* BCMC^®^ 02129, *B. longum* BCMC^®^ 02120) significantly altered the small intestinal microbiome structure and reduced pathogenic bacteria in MASLD patients (123). However, a later clinical study using the same probiotic formulation found no significant effect on hepatic steatosis or fibrosis, although it demonstrated stabilization of intestinal mucosal immune function and enhanced mucosal permeability. This suggests a potential role in improving gut barrier integrity rather than directly regulating hepatic lipid metabolism (124). Another six-strain probiotic blend (*L. acidophilus* CBT LA1, *Lacticaseibacillus rhamnosus* CBT LR5, *Lacticaseibacillus paracasei* CBT LPC5, *Pediococcus pentosaceus* CBT SL4, *B. lactis* CBT BL3, *B. breve* CBT BR3) effectively reduced body weight, body fat, and intrahepatic fat content in adults with MASLD (125). However, other trials showed limited efficacy. In biopsy-confirmed MASH patients, a six-month supplementation with a probiotic containing *L. acidophilus* ATCC SD5212 and *B. lactis* HNO19 was administered. This intervention only mildly improved the aspartate aminotransferase to platelet ratio index (APRI) score, without significantly altering other liver enzymes, inflammatory markers, metabolic parameters, or GM composition (126). Similarly, in obese children, probiotics combined with lifestyle intervention showed limited benefit, with no marked changes in core indicators such as liver function, blood lipids, or glucose metabolism (127). This discrepancy may be attributed to the dynamic developmental state of pediatric GM, which exhibits lower stability and diversity than adult GM. Notably, even in trials without overall significant clinical efficacy, probiotic modulation of specific host pathways can still be observed.

**Table 2:** Clinical studies of probiotics in MASLD/MASH models.

No.	Probiotics	N	Diagnosis	Intervention	Mechanism	Outcomes	GM changes	References
01	HEXBIO^®^	40	CAP ≥ 263 dB/m	6 × 10^9^ CFU/day, 6 months	Modulation of GM, Regulation of gut mucosal immune response and inflammation	IFN-γ, TNF-α ↓; IL-6 ↑	Actinobacteria ↑, Proteobacteria phylum ↓; unclassified_Proteobacteria, unclassified_Streptococcus, unclassified_Stenotrophomonas species ↓; unclassified_Fusobacterium, unclassified_Clostridium species ↑	(123)
02	Six-strain probiotic blend	68	BMI ≥ 25 kg/m^2^ and mean hepatic MRI-PDFF value ≥ 5.0%.	1 × 10^9^ CFU/day, 12 weeks	Modulation of GM, Reduction of inflammation, Improvement of metabolic profile	Body weight, BMI, body fat percentage, MRI-PDFF ↓; TG, TC ↓; TNF-α ↓	*Agathobaculum*, *Dorea* (OTU 527923), *Dorea* (OTU 195044), *Dorea* (OTU 470168), *Blautia*, *Ruminococcus* genus ↑	(125)
03	Eight-strain probiotic blend (*L. paracasei*, *L. plantarum*, *L. acidophilus*, *L. bulgaricus*, *B. longum*, *Bifidobacterium infantis, B. breve*, *S. thermophilus*)	39	liver biopsy-confirmed MASLD Patients	675 billion CFU/day (2 capsules, three times daily), 12 months	Modulation of GM, Enhancing gut barrier function and mitigating the translocation of LPS	ALT, AST, ALP, TBil ↓; TNF-α, IL-1β, IL-6 ↓; LPS ↓; Leptin ↓; NAS ↓	SIBO ↓	(164)
04	Five-strain probiotic blend (*L. casei*, *L. rhamnosus*, *L. acidophilus*, *B. longum*, *B. breve*)	111	Hepatic steatosis ≥ grade II on ultrasonography and ALT > 1.5 × ULN	50 billion CFU/day, 12 weeks	/	TG ↓; ALT, AST, GGT, ALP ↓; hs-CRP ↓	/	(165)

*N*, total number of patients; BMI, body mass index; MRI-PDFF, MRI proton density fat fraction; CAP, controlled attenuation parameter; TBil, total bilirubin; SIBO, small intestinal bacterial overgrowth.

Advances in microbial analytical techniques have spurred extensive exploration of next-generation probiotics. Among these, *Akkermansia muciniphila* has emerged as a research focus due to its inverse association with metabolic disorders. In MASLD animal models, intervention with *A. muciniphila* from the Center of Industrial Culture significantly reshapes the GM—such as promoting butyrate-producing bacteria—improves systemic metabolic parameters, and modulates the BA enterohepatic circulation by differentially regulating hepatic and intestinal FXR expression (119). Notably, pasteurization of *Akkermansia muciniphila* not only preserves but also enhances certain aspects of its beneficial effects on metabolic disorders. This finding may help address the stability and shelf-life challenges faced by live bacterial formulations during storage and transportation (128). Certain members of the *Prevotella* genus exhibit distinctive therapeutic potential. *Bacteroides thetaiotaomicron* strain ATCC 29148 has been found to activate the gut–liver folate metabolism pathway, enhancing the synthesis of S-adenosylmethionine (SAM) and polyunsaturated fatty acids (PUFAs). This dual action cooperatively improves hepatic lipid profiles and insulin sensitivity, thereby attenuating liver steatosis (129). Furthermore, this bacterium specifically upregulates hepatic fatty acid desaturases 1 and 2 (FADS1/FADS2), increasing the proportion of PUFAs while reducing pro-inflammatory monounsaturated fatty acids, which collectively alleviates hepatic lipid accumulation and inflammation (129). Similarly, intervention with *Bacteroides ovatus* (99.6% similarity to *B. ovatus* ATCC 8483) markedly elevates total fecal SCFAs, particularly butyrate and propionate levels. It concurrently upregulates the fatty acid oxidation gene PPARα and downregulates lipogenic genes *Srebf1* and *Fasn*, leading to significant amelioration of hepatic steatosis and inflammation (130).

In summary, while substantial preclinical evidence suggests that oral probiotics can target the GM and exert therapeutic effects on MASLD via the gut–liver axis, current clinical trial results remain controversial. Future studies require standardization in strain selection, dosage, and treatment duration. Notably, findings regarding *A. muciniphila* and *Bacteroides* are predominantly derived from preclinical research. Therefore, more rigorous clinical studies are needed to evaluate the efficacy of specific bacterial strains. Furthermore, associated risk assessments—such as the potential generation of harmful gut metabolites—warrant further attention.

### 3.2 FMT

FMT is currently applied mainly for the treatment of recurrent *Clostridioides difficile* infection and inflammatory bowel disease, both of which share a common pathological feature: disruption of the GM. FMT restores a healthy microbial community, thereby reversing gut dysbiosis and reestablishing microbial homeostasis (131, 132). MASLD is characterized by chronic, multifactorial GM dysbiosis, which contributes to increased translocation of LPS into the circulation, local and systemic inflammation, and insulin resistance. Therefore, FMT holds therapeutic potential for MASLD by fundamentally reshaping the host’s metabolic microenvironment, although current supporting evidence remains limited.

In preclinical studies, the benefits of FMT have been demonstrated in MASLD mouse models (Table 3). One study showed that multi-donor FMT significantly enriched beneficial bacterial taxa, such as *Eubacterium limosum* and *Akkermansia*, while markedly improving metabolic parameters and restoring intestinal barrier integrity (133). Furthermore, another animal experiment revealed that FMT from healthy donor mice alleviated HFD-induced steatohepatitis, as evidenced by reduced hepatic steatosis and inflammation. The treatment also reestablished microbial homeostasis, increasing the relative abundance of beneficial bacteria, including *Christensenellaceae* and *Lactobacillus*. Additionally, FMT enhanced butyrate production, strengthened gut barrier function, and mitigated the severity of endotoxemia (13).

**Table 3:** Preclinical studies on FMT in MASLD/MASH models.

No.	Donor	Recipient	Intervention	Mechanism	Outcomes	GM changes	References
01	Normal diet mice	HFD-induced MASH mice	Daily gavage with 200 μL fecal suspension, 8 weeks	Modulation of GM and microbial metabolites, Enhancement of hepatic fatty acid oxidation and cholesterol clearance, Suppression of *de novo* lipogenesis, Enhancement of intestinal barrier, Amelioration of systemic and hepatic inflammation	Body weight, liver index ↓; serum ALT, AST ↓; hepatic TG, TC ↓; NAS ↓; TNF-α, IL-17, IFN-γ, IL-6 ↓; Foxp3, IL-4, IL-22 ↑	*Lactobacillus*, *Christensenellaceae* genus ↑; *Odoribacter*, *Oscillibacter* genus ↓	(13)
02	Healthy human	HFD-induced MASLD mice	Daily gavage with 200 μL fecal suspension, 12 weeks	Modulation of GM, Restoration of intestinal barrier integrity, Suppression of hepatic inflammation	Body weight ↓; NAS ↓; TG, TC, LDL-C ↓; ALT ↓; LPS ↓; Occludin, Claudin-1, E-cadherin ↑	Bacteroidetes phylum ↑; Proteobacteria phylum ↓; Lachnospiraceae, Muribaculaceae family ↑; *Blautia*, *Akkermansia*, *Lachnospiraceae_*NK4A136_group, *Eubacterium*_fissicatena_group genus ↑	(133)
03	Normal diet mice	HFHCF-induced MASH mice	Daily gavage with 200 μL fecal suspension, once every 2 days, 8 weeks	Modulation of GM, Enhancement of intestinal barrier function, Activation of hepatic antioxidant pathway, Suppression of hepatic inflammation and fibrosis, Modulation of microbial metabolic balance	Serum TNF-α, IL-6 ↓; 4-HNE ↓; LPS ↓; α-SMA, Fibronectin ↓; NRF2 ↑	*Facklamia*, *Aerococcus* genus ↓; *Clostridium* genus ↑	(166)

HFHCF, high fat, high cholesterol, and fructose; Foxp3, forkhead box protein P34-HNE: 4-hydroxynonenal; α-SMA, α-smooth muscle actin; NRF2, nuclear factor erythroid 2-related factor 2.

Multiple clinical studies have indicated that FMT alleviates hepatic steatosis in patients with MASLD (Table 4). FMT derived from healthy donors effectively reduced hepatic fat accumulation and restored gut microbial balance, notably by decreasing the F/B ratio and enriching butyrate-producing bacteria (134). Notably, this study found that FMT outperformed probiotics in improving both hepatic fat content and GM composition. In a study by Witjes et al. (135), fecal microbiota from four healthy, lean vegetarian donors were transplanted into MASLD patients via a nasoduodenal tube. Following transplantation, patients exhibited improvements in biochemical liver function and histological necroinflammatory scores, including reductions in lobular inflammation and hepatocellular ballooning. However, no significant changes in the degree of hepatic steatosis or fibrosis were observed. Additionally, post-FMT analysis revealed increased abundances of *Ruminococcus*, *Clostridium hathewayi*, *Faecalibacterium*, and *Prevotella* in the GM of recipients. In contrast, a recent randomized, double-blind, controlled trial demonstrated that neither consecutive allogeneic nor autologous FMT significantly altered hepatic fat content in MASLD patients (136). This discrepancy may be attributable to the high baseline GM diversity among patients, which limits colonization by exogenous microbes. Furthermore, the complex, multifactorial pathophysiology of MASLD, combined with the relatively small sample size, may also have influenced the study outcomes. For MASLD patients, allogeneic FMT, compared to autologous FMT, significantly modulated hepatic DNA methylation patterns, enriched beneficial bacterial taxa, and improved plasma metabolite profiles (137). Nevertheless, neither intervention led to significant improvements in insulin resistance or hepatic fat accumulation (138). Similarly, another study found that although allogeneic FMT reduced small intestinal permeability at 6 months, it did not result in notable amelioration of hepatic steatosis (138).

**Table 4:** Clinical studies on FMT in MASLD/MASH models.

No.	Donor	Recipient	N	Intervention	Mechanism	Outcomes	GM changes	References
01	Healthy human	Patients with MASLD based on CAP (moderate)	75	200 mL/day × 3 days (colonoscopy and Enemas)	Modulation of GM and its metabolic functions	CAP ↓	F/B ↓; Bacteroidetes phylum ↑; Proteobacteria phylum ↓; *Escherichia–Shigella* genus ↓	(134)
02	Lean vegan donors (BMI < 25)	Obese, treatment-naïve individuals with hepatic steatosis on ultrasound (biopsy-proven MASLD)	10	Administered via gastro-duodenoscopy (week 0) and CORTRAK^®^-guided duodenal tube (weeks 8 and 16)	Modulation of GM and metabolic functions, Modulation of hepatic gene expression involved in inflammation and lipid metabolism	GGT ↓; ARHGAP18, SDS ↑; phenylacetylglutamine, isoleucine ↑; phenyllactic acid ↓	*Ruminococcus*, *Eubacterium hallii*, *Faecalibacterium*, *Prevotella* genus ↑; *Lachnospiraceae-related bacteria* genus ↓	(135)
03	Lean vegan donors	Treatment-naïve, obese individuals with hepatic steatosis on ultrasound and biopsy-proven NAFLD	21	Administered via gastro-duodenoscopy (week 0) and CORTRAK^®^-guided duodenal tube (weeks 8 and 16)	Modulation of GM; Alteration of plasma metabolites; Regulation of hepatic DNA methylation	PAC, PAG ↑; Multiple CpG sites differentially methylated (e.g., *TARS*, *ZFP57*)	*Blautia*, *Eubacterium* genus ↑; *Lactobacillus* genus ↓	(137)

ARHGAP18, Rho GTPase activating protein 18; SDS, serine dehydratase; PAC, phenylacetylcarnitine; PAG, phenylacetylglutamine.

In conclusion, FMT holds substantial therapeutic potential and scientific value for the treatment of MASLD. Although variability in clinical outcomes persists, likely due to differences in sample sizes, study designs, and routes of administration, the overall body of evidence supports its beneficial role in MASLD management. Future research should focus on identifying specific beneficial bacterial strains with superior colonization capacity, tailored to patients with diverse geographic, ethnic, and dietary backgrounds, thereby promoting a resilient and balanced gut microenvironment. Enhancing the delivery efficiency of viable bacteria and improving their colonization success will remain critical priorities for future investigation.

### 3.3 Natural products

Although research on MASLD has attracted growing attention in recent years, traditional Chinese medicine records from ancient times have already described the alleviation of related symptoms such as hypochondriac pain and abdominal distension. Modern pharmacological studies have revealed that the active constituents of these traditional remedies primarily originate from plants, including phenolic compounds, polysaccharides, and other natural products. These bioactive components exhibit lipid-lowering, anti-inflammatory, and GM–modulating properties, as well as effects on microbial metabolites (Table 5).

**Table 5:** Preclinical studies on natural products in MASLD/MASH models.

No.	Natural products	Model	Intervention	Mechanism	Outcomes	GM changes	References
Polyphenols							
01	Nobiletin	HFHS-induced MASLD mice	100 mg/kg/day, 12 weeks	GM Modulation, Enhancement of hepatic fatty acid oxidation and cholesterol clearance via BA synthesis, and suppression of *de novo* lipogenesis	Body weight ↓; Liver weight ↓; Serum ALT, AST ↓; Serum and liver TG, TC ↓; NAS ↓; MA ↑; PPARα ↑; Cyp7a1 ↑	Bacteroidetes phylum ↑; Firmicutes, Verrucomicrobia, Proteobacteria phylum ↓; *Allobaculum*, *Lactobacillus* genus ↑	(139)
02	Caffeic acid phenethyl ester	HFD-induced MASLD mice	75 mg/kg/day, 8 weeks during prevention and 8 weeks post-modeling during treatment	GM Modulation, inhibition of BSH/FXR signaling and intestinal ceramide synthesis, promotion of GLP-1 secretion, and amelioration of hepatic lipid metabolism	Body weight ↓; Liver weight ↓; Hepatic TG ↓; SREBP-1c, *Fasn*, Acc1, Scd1, Cd36 ↓; TNF-α, IL-1β, PAI1, NLRP3 ↑; Intestinal FXR	Firmicutes, Parabacteroides phylum ↓; Actinobacteria phylum ↑; *Bacteroides*, *Enterococcus*, *Bilophila*, *Helicobacter* genus ↑; *Intestinimonas*, *Lachnospiraceae*, *Parabacteroides*, *Faecalibacterium* genus ↓	(167)
03	flavonoid extract from *Smilax glabra Roxb*	HFD-induced MASLD mice	300 mg/kg/day, 12 weeks	GM Modulation, Increased SCFA production, and improvement of hepatic lipid metabolism and oxidative stress	Liver weight, Liver-to-body weight ratio ↓; Serum and liver TC, TG, FFA ↓; Serum ALT, AST ↓; Hepatic MDA ↓; SOD ↑	F/B, Verrucomicrobia phylum ↓; *Muribaculaceae*, *Bacteroidetes*, *Alloprevotella* genus ↑; *Akkermansia*, *Phaseolartobacterium*, *Lactobacillale* genus ↓	(140)
04	Pure total flavonoids from citrus	HFD-induced MASH mice	50 mg/kg/day, 12 weeks	GM Modulation, Regulation of BA metabolism, and activation of hepatic FXR/TGR5	ALT, AST ↓; TC ↓; NAS ↓; Toxic BAs ↓; FXR, TGR5 ↑	F/B ↓; Christensenellaceae, Erysipelotrichaceae, Bacteroidaceae family ↑; Porphyromonadaceae, Streptococcaceae family ↓; A*llobaculum*, *Bacteroides*, *Akkermansia* genus ↑; *Eubacterium* genus ↓	(141)
05	Quinoa bran polyphenol extract	HFD-induced MASLD mice	400 mg/kg/day, 10 weeks	Modulation of GM and lipid metabolism, amelioration of oxidative stress and inflammation, Regulation of hepatic metabolites, and activation of the AMPK signaling pathway	Body weight, Liver weight ↓; Serum TC, TG, LDL-C ↓; ALT, AST ↓; CAT, SOD, GSH-Px ↑; MDA ↓; IL-1β, IL-6, TNF-α ↓; Acetate, Propionate, Butyrate ↑	Firmicutes phylum ↓; *Faecalibaculum*, *Erysipelatoclostridium*, *Ruminococcus_*gnavus_group, C*lostridium*_sensu_stricto_13 genus ↑; *Dubosiella*, *Blautia* genus ↓	(142)
06	Theabrownin	HFD-induced MASLD mice	2,300 mg/kg/day, 14 weeks	GM Modulation, Elevation of serotonin levels in the circulation and white visceral adipose tissue, Reduction of hepatic serotonin, inhibition of the hepatic HTR2A/PPARα	Body weight ↓; Serum TG, TC, LDL-C ↓; ALT, AST ↓; ROS, MDA ↓; SOD, CAT ↑	Verrucomicrobiota phylum ↑; *Akkermansia*, *Bacteroides*, *Parabacteroides* genus ↑	(143)
07	Chlorogenic Acid	HFD-induced MASLD mice	60 mg/kg/day by oral gavage, co-treatment with HFD, 12 weeks	GM Modulation, Improvement of intestinal barrier function, Suppression of hepatic inflammation, and amelioration of lipid metabolism and insulin resistance	NAS ↓; Serum ALT, AST ↓; FBG, HOMA-IR ↓; TC, TG ↓; TLR4 ↓; GLP-1 ↑; Occludin, ZO-1 ↑; LPS ↓	*Bifidobacterium* genus ↑, *E. coli* genus ↓	(144)
Polysaccharides							
08	*Prunella vulgaris* L. polysaccharides	CDAHFD -induced MASH mice	200 mg/kg/day, 6 weeks	GM Modulation, Improvement of intestinal barrier function, alleviation of hepatic lipid accumulation, inflammation, and fibrosis	Serum ALT, AST ↓; Hepatic TG, NEFA ↓; NAS ↓; ZO-1 ↑; Hepatic NLRP3 ↑; α-SMA + cells ↑	Actinomycetes phylum ↑; Proteobacteria phylum ↓; Clostridiaceae, Helicobacteraceae family ↓; Coriobacteriaceae family ↑; *Bacteroides* genus ↓; *Adlercreutzia*, *Roseburia*, *Bacteroides* genus ↑	(168)
09	Polysaccharides from *Lanzhou Lily*	HFD-induced MASLD mice	Low: 100 mg/kg/day; High: 200 mg/kg/day by oral gavage, 4 weeks	Modulation of GM and associated metabolic pathways, Improvement of lipid metabolism, attenuation of hepatic steatosis, and suppression of liver inflammation	Body weight, Liver weight, Liver-to-body weight ratio ↓; Serum TG, TC, LDL-C ↓; Hepatic steatosis ↓; Hepatic TNF-α, IL-1β ↓	F/B ↓; Bacteroidota ↑, Firmicutes, Verrucomicrobiota, Desulfobacterota phylum ↓; *Alistipess*, *Aerococcus*, *Dubosiella*, *Lachnospiraceae_*NK4A136_group genus ↑; *Turicibacter*, *Bifidobacterium*, *Akkermansia*, *Colidextribacter*, *Prevotellaceae-*UCG-001 genus ↓	(169)
10	Tegillarca granosa Polysaccharide	HFD-induced MASLD mice	Low: 200 mg/kg/day; High: 400 mg/kg/day, 16 weeks concurrently with HFD	GM Modulation, Attenuation of hepatic steatosis, oxidative stress, and inflammation; Activation of the AMPKα1/PPAR-α/CPT1A, Suppression of SREBP-1c/FAS/HMGCR expression, Improvement of systemic lipid metabolism, Increased SCFA production	Body weight ↓; Serum TC, TG, LDL-C ↓; ALT, AST ↓; Hepatic TC, TG, NEFA ↓; Hepatic SOD, GSH-Px ↑; MDA ↓; IL-6, TNF-α ↓	Firmicutes, Actinobacteriota, Desulfobacterota phylum ↓; Bacteroidota phylum ↑; *Lactobacillus*, *Lachnospiraceae_*NK4A136_group genus ↑; *Faecalibaculum*, *Desulfovibrio*, *Bifidobacterium*, *Enterorhabdus*, *Monoglobus* genus ↓	(170)
11	*Ophiopogon japonicus* polysaccharide (MDG)	HFD-induced MASLD mice	MDG-1: HFD + 5‰ MDG-1; MDG-C: HFD + 8% MDG-C, 12 weeks	Modulation of GM composition, diversity, and intestinal barrier integrity, increased SCFA production, Inhibition of lipogenesis and promotion of fatty acid oxidation gene; suppression of hepatic inflammation	Body weight ↓; Liver weight, Liver-to-body weight ratio ↓; AST, ALT ↓; Hepatic TC, TG ↓; IL-1β, IL-6, TNF-α ↓; IL-10 ↑; Occludin, ZO-1, Muc2 ↑	F/B ↓; *Akkermansia*, *Lachnospiraceae*_NK4A136_group, *Alistipes*, *Lactobacillus*, *Roseburia*, *Turicibacter* genus ↑; *Lactococcus*, *Enterorhabdus*, *Erysipelatoclostridium* genus ↓	(171)
12	Yellow tea polysaccharides	HFD-induced MASLD mice	400 mg/kg/day, 12 weeks	GM Modulation, Inhibition of the ileal FXR-FGF15 axis and activation of the hepatic FXR-SHP pathway, Enhanced BA excretion, attenuation of hepatic steatosis and inflammation	Body weight, Liver weight, Liver-to-body weight ratio ↓; Serum and Hepatic TC, TG ↓; Serum ALT, AST ↓; Cyp7a1, Cyp27a1 ↑; Cyp8b1 ↓	F/B ↓; Verrucomicrobia phylum ↑; *Lactobacillus*, *Streptococcus*, *Clostridium*, *Bifidobacterium*, *Burkholder*, *Turicibacter* genus ↓; *esulfovibrionacea* genus ↑	(148)
13	PAMK	Western diet + low-dose CCl₄-induced MASH mice	700 mg/kg/day, 12 weeks	GM Modulation, Improvement of serum phospholipid profile, Attenuation of hepatic steatosis, inflammation, and fibrosis	Body weight, Liver weight ↓; Hepatic TC, TG ↓; Serum ALT, AST ↓; FBG ↓; NAS ↓; Hepatic TNF-α, IL-6, MCP-1, IL-18, IL-1β ↓	F/B ↓; *Faecalibaculum_rodentium*, *aecalibaculum* genus ↓; *Muribaculaceae* genus ↑	(150)
14	*Lycium barbarum* polysaccharide	HFD-induced MASLD mice	50 mg/kg/day, 8 weeks	GM Modulation, Increased SCFA production, Restoration of intestinal barrier integrity, Inhibition of hepatic TLR4/NF-κB	Serum and Hepatic TC, TG ↓; Serum LDL, FFA ↓; NAS ↓; FBG, HOMA-IR ↓; IL-6, TNF-α, IL-1β, MCP-1 ↓; IL-10 ↑; ZO-1, Occludin ↑; LPS ↓	F/B ↓; Deferribacteres phylum ↑; Deferribacteraceae, family ↑; Enterococcaceae family ↓	(151)
15	Polysaccharides Extracted from Old Stalks of *Asparagus officinalis* L	HFD-induced MASLD mice	50 mg/kg/day, 8 weeks	GM Modulation, Activating AMPK/SREBPs to inhibit lipogenesis; Restoration of intestinal barrier integrity; Suppression of TLR4/NF-κB	Serum TC, TG, LDL-C ↓; Hepatic TC, TG ↓; ALT, AST ↓; Butyrate ↑; LPS ↓; TNF-α, IL-6 ↓	Firmicutes phylum ↑; Bacteroidetes, Proteobacteria phylum ↓; Lachnospiraceae family ↑; *Roseburia, Rikenella* spp. genus ↑; *Escherichia–Shigella* genus ↓	(152)
16	APS	HFD-induced MASLD mice	4% APS mixed in HFD, 8 weeks	GM Modulation, Promotion of acetate production, Inhibition of hepatic lipogenesis, Promotion of fatty acid oxidation	Body weight, fat index ↓; Hepatic TG ↓; Hepatic steatosis score ↓; Serum ALT ↓; *Fasn* ↑	F/B ↓; *Desulfovibrio*, *Parabacteroides*, *Acetatifactor*, *Alistipes* genus ↑	(149)
Saponins							
17	Soyasaponin	MCD diet-induced MASH mice	80 μmol/kg/day, 16 weeks	GM Modulation, Restoration of intestinal barrier integrity, Modulation of BA metabolism	NAS ↓; Serum ALT, AST ↓; Hepatic TG, TC ↓; Hepatic TNF-α, IL-6 ↓; Occludin, ZO-1 ↑; Unconjugated BAs ↓; Secondary BAs ↑; LPS ↓	Firmicutes phylum ↓; Verrucomicrobia phylum ↑; Erysipelotrichaceae, unidentified_Clostridiales, Eggerhellaceae, Atopobiaceae family ↓; Akkermansiaceae family ↑; *Akkermansia*, *Roseburia* genus ↑; *Rikenella*, *Aerococcus*, *Jeotgalicoccus*, *Gemella*, *leibacteriu* genus ↓	(153)
18	Glycyrrhizic acid	HFD-induced MASLD mice	40 mg/kg/day, 12 weeks	GM Modulation, Suppression of microbial carbohydrate metabolism	Body weight ↓; Hepatic TG, TC ↓; Hepatic lipid vacuoles ↓	Lachnospiraceae, Coriobacteriaceae family ↓; Peptostreptococcaceae family ↑; *Blautia*, *Collinsella* genus ↓; *Rombousia*, *Turicibacter* genus ↑	(154)
19	PNS	HFD-induced MASLD mice and genetic ob/ob mice	800 mg/kg/day by oral gavage for 8 weeks after 4-week modeling	GM Modulation, Enhancement of Intestinal barrier, Inhibition of the TLR4/MyD88; Reduction in gut-derived SCFA translocation to the liver, Activation of the AMPKα, Regulation of hepatocellular lipid metabolism	Body weight ↓; Serum and hepatic TG, TC, FFA, LDL-C ↓; Serum AST, ALT ↓; FBG ↓; Hepatic steatosis ↓; TNF-α, IL-6 ↓	F/B ↓; *Parabacteroides distasonis* genus ↑	(155)
20	Gypenosides (GP)	HFHC diet and fructose water-induced MASLD mice	300 mg/kg, 6 weeks	GM Modulation, insulin sensitivity improvement, regulation of lipid metabolism genes, and antioxidant/anti-inflammatory effects	Body weight, Liver weight ↓; Serum ALT, AST, TG ↓; HOMA-IR ↓; Hepatic steatosis and inflammation ↓; CD36, ACC1, PPARγ, SOD ↓	F/B ↓; *Fissicatena*, *Akkermansia* genus ↓	(172)
Alkaloids							
21	*Ramulus mori* (Sangzhi) alkaloids	HFD-induced MASLD mice	200 mg/kg/day, 6 weeks	GM Modulation, Enhancement of the intestinal barrier, Regulation of lipid and bile acid metabolism, Improvement of metabolism and inflammation	Body weight ↓; Serum TC, LDL ↓; EPA, DHA, α-linolenic acid, lithocholic acid ↑	F/B ↓; Verrucomicrobacteria, Actinobacteria phylum ↑; *Akkermansia*, *Bifidobacterium* genus ↑	(157)
22	Sinapine	HFD-induced MASLD mice	500 mg/kg, 12 weeks	GM Modulation, Promotion of SCFAs production, Activation of GPR43, Suppression of inflammation, Improvement of insulin sensitivity and lipid metabolism	Body weight ↓; Serum TG, LDL-C ↓; Hepatic AST, ALT, V-LDL ↓; FBG, HOMA-IR ↓; NAS ↓; Intestinal NF-κB, TNF-α ↓; Adipose tissue TNF-α, IKK ↓; IRS-1 ↑	F/B ↓; Proteobacteria phylum ↓; Akkermansiaceae, Lactobacillaceae family ↑; Lachnospiraceae, Erysipelotrichaceae family ↓; *Blautia*, *Akkermansia*, *Lactobacillus*, *Bifidobacterium* genus ↑; *Lachnoclostridium*, *Romboutsia*, *Roseburia*, *Clostridium* genus ↓	(158)
23	EPI	MCD diet-induced MASH mice	50, 100, 200 mg/kg/day, 4 weeks	GM Modulation, Activation of SHP, and subsequent inhibition of the SREBP-1/FASN pathway	Serum ALT, AST ↓; Hepatic TC, TG, MDA ↓; Serum hyaluronic acid ↓; NAS ↓; IL-6, IL-1β, TNF-α mRNA ↓; IL-1β, TGF-β ↓; CD68 ↓	Bacteroidetes, Proteobacteria, Firmicutes family ↑; *Akkermansia*, *Prevotella* ↑; *Sutterella*, *Adlercreutzia* ↓	(159)
Terpenes							
24	Bentong ginger oleoresin	HFD-induced MASLD mice	130 mg/kg/day, 14 weeks	GM Modulation, Regulation of lipid metabolism, Inhibition of inflammation and oxidative stress, Enhancement of mitochondrial function	Body weight, Liver weight ↓; Serum TC ↓; Hepatic TC, TG ↓; TNF-α, IL-6, IL-1β ↓; MDA ↓; CS, SDH, NADH ↑	F/B ↓; *Lachnospiraceae_*NK4A136_group, *Fournierell* genus ↓; *Lactobacillus*, *Roseburia*, *Odoribacter*, *Agathobacter* genus ↑	(160)

HFHS, high-fat, high-sucrose; ACC1, acetyl-CoA carboxylase 1; PAI1, plasminogen activator inhibitor-1; FFA, free fatty acid; CAT, catalase; GSH-Px, glutathione peroxidase; HTR2A, 5-hydroxytryptamine receptor 2A; CDAHFD, choline deficiency, L-amino acid-defined high-fat diet; HMGCR, 3-hydroxy-3-methylglutaryl coenzyme A reductase; CS, citrate synthase; SDH, succinate dehydrogenase; EPA, eicosapentaenoic acid; DHA, docosahexaenoic acid; IKK, IκB kinase; IRS-1, insulin receptor substrate 1; CD68, cluster of differentiation 68; NADH, nicotinamide adenine dinucleotide.

#### 3.3.1 Phenolic

Phenolic compounds are characterized by a benzene ring with phenolic hydroxyl groups. Their conjugated systems enhance electron delocalization, leading to the formation of subclasses such as flavonoids, lignans, and stilbenes. These structural features confer biological activities, including free radical scavenging, metal ion chelation, and modulation of cellular signaling pathways. Consequently, they contribute to the amelioration of MASLD by reducing hepatic lipid accumulation and suppressing chronic inflammation.

Animal studies have provided mechanistic insights into these effects. Nobiletin was shown to modulate GM in MASLD mice, leading to upregulation of myristoleic acid (MA) metabolism, thereby directly reducing hepatic lipid accumulation and improving glucose metabolism (139). Flavonoid extracts from *Smilax glabra Roxb* were metabolized by the gut flora into aglycones and further degraded into phenolic acids, lowering the F/B ratio and promoting SCFA production (140). In an HFD-induced mouse model of MASH, citrus flavonoids enriched specific gut microbes, facilitated the conversion of primary to secondary BAs, and activated the hepatic FXR/TGR5 pathway, thereby alleviating primary BA-induced liver injury (141). Furthermore, metabolomic analysis in MASLD mouse models treated with quinoa bran polyphenol extract revealed significant upregulation of the anti-inflammatory allyl isothiocyanate along with an AMPK-activating metabolite. Concurrently, markers of dysglycemia, including sucrose and isomaltose, were downregulated. These changes collectively suppressed hepatic gluconeogenesis, reduced precursors for fatty acid synthesis, and ultimately lowered liver fat content (142). Theabrownin, a polymeric polyphenolic pigment derived from oxidized tea polyphenols, exhibits low oral bioavailability (<5%) and primarily depends on β-glucosidase produced by *Bacteroides* for hydrolysis into oligomeric phenolic acids. Animal studies indicate that these metabolites accumulate in the liver and adipose tissues, promote fatty acid oxidation and adipose tissue lipolysis, and inhibit oxidative stress pathways, thereby mitigating hepatic steatosis and oxidative damage (143). Chlorogenic acid enhances fiber fermentation by *Bifidobacterium*, thereby increasing SCFA production. This process stimulates GLP-1 secretion from intestinal L cells, thereby improving insulin sensitivity, enhancing gut barrier integrity, and reducing LPS translocation into the portal circulation (144). Additionally, other phenolic compounds, including curcumin (145), naringin (146), and quercetin (147), have also been reported to ameliorate MASLD and associated gut dysbiosis.

#### 3.3.2 Polysaccharides

Polysaccharides are high-molecular-weight polymers composed of monosaccharide units linked by glycosidic bonds. Their structural diversity arises from variations in monomer composition and linkage patterns, primarily including homopolysaccharides, heteropolysaccharides, and structurally modified polysaccharides. The intricate chain architectures and specific sugar sequences of these compounds enable effective modulation of the GM. Polysaccharides containing sulfate groups and uronic acid components exhibit strong antioxidant activity, scavenging excess free radicals in the liver and alleviating oxidative stress. Meanwhile, the high viscosity of certain heteropolysaccharides can delay nutrient absorption, thereby improving lipid metabolism disorders and insulin resistance.

A substantial body of animal studies confirms that polysaccharides ameliorate MASLD through the following mechanisms. First, polysaccharides can modulate the relative abundance of beneficial versus pathogenic GM and restore overall microbial diversity. For instance, yellow tea polysaccharides have been shown to restore GM homeostasis by upregulating *Akkermansia* and the Desulfovibrionaceae family while downregulating *Lactobacillus* and *Streptococcus*, thereby improving gluco-lipid metabolism and exerting protective effects against MASLD in animal models (148). Similarly, A*stragalus* polysaccharides (APS) have been demonstrated to enrich beneficial GM and downregulate the F/B ratio (149). Second, polysaccharides improve insulin sensitivity, further suppressing hepatic *de novo* lipogenesis, alleviating inflammation, and delaying the progression of liver fibrosis. Polysaccharides from *Atractylodes Macrocephala* Koidz (PAMK) significantly reduced FBG and insulin levels, improved the insulin sensitivity index, and enhanced glucose tolerance in a mouse model of MASH induced by a high-fat/high-sugar diet (HFSD) combined with low-dose carbon tetrachloride (150). Similarly, *Lycium barbarum* polysaccharides also improved insulin sensitivity in MASLD mice while markedly decreasing serum and hepatic levels of TC and TG (151). Third, polysaccharides enhance intestinal barrier function. Polysaccharides from asparagus stems, characterized by low uronic acid content, enhance binding to the intestinal mucus layer and upregulate the expression of the tight junction protein occludin. This mechanism reduces intestinal permeability, promotes barrier repair, and ultimately lowers plasma LPS levels (152).

#### 3.3.3 Saponins

Saponins are amphiphilic secondary metabolites composed of a hydrophobic sapogenin linked to a hydrophilic sugar moiety via glycosidic bonds. The main subclasses include triterpenoid saponins and steroidal saponins. Due to their inherent structure, saponins exhibit low oral bioavailability and rely mainly on biotransformation by GM-derived β-glucosidases. This hydrolysis yields active metabolites such as secondary aglycones and SCFAs.

GM-mediated biotransformation is central to the anti-MASLD effects of saponins. In a MCD diet-induced mouse model of MASH, soyasaponin intervention significantly reduced serum levels of ALT, AST, TC, and TG, while improving intestinal barrier integrity and microbial structure. Notably, these beneficial effects were abolished upon antibiotic-induced depletion of the GM, demonstrating that an intact GM is essential for soyasaponin activity (153). Other saponins ameliorate MASLD through similar mechanisms. Glycyrrhizic acid markedly lowered hepatic TC and TG content, alleviated histopathological liver injury, restored microbial α-diversity, and increased the proportion of beneficial GM in MASLD mice (154). *Panax notoginseng* saponins (PNS) exhibited hepatoprotective effects in a MASLD mouse model, primarily by inhibiting the TLR4-mediated inflammatory signaling pathway and enhancing gut barrier function (155). Ginsenoside Rg5 promoted the proliferation of beneficial bacteria such as the Bacteroidetes phylum and *Akkermansia* while reducing the relative abundance of harmful bacteria. Furthermore, it activated the LKB1/AMPK/mTOR signaling pathway to stimulate energy metabolism, collectively hindering MASLD progression (156).

#### 3.3.4 Alkaloids

Alkaloids are nitrogen-containing basic natural products in which the core nitrogen atom can be embedded in aliphatic, aromatic, or heterocyclic ring systems, giving rise to structurally diverse subclasses. Several alkaloids have shown potential in ameliorating MASLD by modulating GM composition and regulating metabolic pathways. *Ramulus mori* (Sangzhi) alkaloids, a polyhydroxy alkaloid preparation extracted from mulberry twigs, approved for treating type 2 diabetes, were found to activate the metabolic pathways of unsaturated fatty acids and BA. They significantly increased the abundance of *Akkermansia* and *Bifidobacterium* and restored intestinal barrier integrity (157). Sinapine, another alkaloid, modulates GM composition and enhances SCFAs production, which in turn activates the GPR43 signaling pathway, suppresses inflammatory cytokine expression, and improves insulin resistance (158). In a mouse model of MCD-diet-induced MASH, Epiberberine (EPI) intervention exhibited dose-dependent effects. Low, medium, and high doses all reduced hepatic lipid accumulation by upregulating SHP and inhibiting the SREBP1/FASN pathway. However, only the high-dose group significantly decreased the liver-to-body weight ratio and showed the most pronounced improvement in hepatic lipid accumulation (159).

#### 3.3.5 Terpenoids

Terpenoids are a class of natural compounds formed through the polymerization of isoprene units. Characteristics such as high lipophilicity and large molecular weight contribute to their low oral bioavailability. To exert their full biological activity, these compounds typically require structural modification by the GM, involving hydrolysis of glycosyl groups or alteration of the core scaffold, thereby generating metabolites with greater membrane permeability and biological potency. Recent studies have shown that terpenoids ameliorate metabolic disorders by modulating the gut microenvironment. For instance, treatment with Bentong ginger oleoresin restored GM balance, as indicated by a reduced F/B ratio. It also downregulated the abundance of *Lactobacillus*, which is associated with SCFA production. This decrease in microbial energy harvest from the diet ultimately contributed to improved lipid metabolism (160).

Research on natural products has predominantly focused on preclinical studies. However, in a 4-month randomized placebo-controlled clinical trial, Ni et al. demonstrated that resistant starch supplementation effectively reduced intrahepatic triglyceride content (IHTC) by an absolute 9.08%. This effect was closely associated with alterations in the GM (161). Future translational research should prioritize clinical trials of natural products to validate these preclinical findings and develop GM-targeted therapies for MASLD.

## 4 Discussion

The role of the GM in MASLD is increasingly being investigated. Both microbial components and their metabolites contribute, directly or indirectly, to the development and progression of MASLD. This article discusses the mechanisms by which GM and their metabolites contribute to MASLD pathogenesis and summarizes the potential applications of probiotics, FMT, and natural products in its treatment. Current evidence indicates that GM dysbiosis drives hepatic steatosis, inflammation, and fibrosis by impairing intestinal barrier function and altering the balance of microbial metabolites, such as SCFAs, BAs, and tryptophan derivatives. The interventions mentioned above exert hepatoprotective effects through multiple pathways, including restoring microbial homeostasis, enhancing barrier integrity, and regulating metabolic processes.

Although current evidence strongly supports the therapeutic potential of targeting the GM for MASLD, significant challenges remain. The efficacy of probiotics, FMT, and natural products has been inconsistent. In the case of probiotic administration, numerous animal studies have demonstrated that specific strains can significantly ameliorate hepatic steatosis and inflammation through multiple mechanisms. However, results from clinical studies have been highly variable. This discrepancy may be attributed to considerable heterogeneity in clinical trial designs, including differences in probiotic formulations, dosages, diagnostic criteria, patient characteristics, and outcome measures. Furthermore, the baseline GM composition of the host may influence the intervention outcomes. Importantly, most clinical trials have provided limited insight into the mechanisms by which probiotics exert their effects in MASLD patients, particularly regarding their impact on microbial composition, intestinal integrity, and microbial metabolites. Future research should focus on identifying next-generation probiotics, such as *Akkermansia*, with confirmed efficacy; elucidating the mechanisms of their functional components, such as outer membrane proteins and extracellular polysaccharides; and developing personalized probiotic therapies.

FMT also has several limitations. While a few preliminary clinical studies have found that FMT can improve hepatic inflammation and insulin sensitivity, recent randomized controlled trials have shown that its effect on reducing hepatic fat content is limited, and its therapeutic benefits are often transient. The efficacy of FMT is strongly influenced by multiple factors, including donor selection, administration route, and recipient microbial colonization resistance. Furthermore, long-term safety concerns and the lack of standardized preparation protocols remain critical barriers to its clinical translation. Future research should prioritize the development of standardized, safe FMT formulations that incorporate rigorous donor screening. Exploring strategies to enhance microbial engraftment through combination therapies involving dietary or pharmacological interventions, as well as utilizing synthetic microbial communities as alternatives to complex fecal mixtures, represents a promising direction for future research.

Natural products demonstrate unique advantages in MASLD management due to their multi-target effects, favorable safety profiles, and diverse sources. Although these compounds often exhibit low oral bioavailability because of their large molecular size or high lipophilicity, they can exert beneficial effects indirectly by modulating the structure and function of the GM. However, current research remains predominantly confined to animal studies, with limitations including unidentified active components, insufficient mechanistic insights, and a lack of clinical translation evidence. Future investigations should integrate metabolomics and metagenomics technologies to elucidate the interplay among natural products, the GM, and the host. Additionally, novel drug delivery systems should be employed to enhance targeting efficiency and bioavailability, while well-designed clinical trials are urgently needed to validate their therapeutic efficacy.

In summary, targeting the GM offers innovative therapeutic strategies for the management of MASLD. However, the three main approaches—probiotics, FMT, and natural products—remain in the translational phase between basic research and clinical application. These interventions share common challenges, including inconsistent efficacy, insufficient standardization, and individual host variations. Future research should aim to elucidate the underlying mechanisms of microbial modulation, establish precise microbiota-based intervention frameworks, and advance the development of personalized treatment strategies. Carefully designed clinical trials are essential to validate the long-term safety and efficacy of these approaches, ultimately leading to breakthroughs in MASLD prevention and treatment. Furthermore, while most current studies focus on the relationship between GM and host metabolism, limited attention has been given to the role of genetic background in microbe-host interactions. Future investigations should also explore how GM and host genetic factors interact to modulate MASLD risk.
